# Association of 24-hour movement guideline adherence, mental health and quality of life in young adults: the role of e-Health literacy

**DOI:** 10.3389/fpubh.2024.1344718

**Published:** 2024-05-22

**Authors:** Lixin Lin, Wei Liang, Runbin Wang, Ryan E. Rhodes, Huaxuan Liu

**Affiliations:** ^1^Physical Education School, Putian University, Putian, Fujian, China; ^2^School of Physical Education, Shenzhen University, Shenzhen, Guangdong, China; ^3^School of Physical Education and Sport Science, Fujian Normal University, Fuzhou, Fujian, China; ^4^School of Exercise Science, Physical and Health Education, University of Victoria, Victoria, BC, Canada

**Keywords:** college student, e-Health literacy, mental health, physical activity, quality of time, screen time, sleep

## Abstract

**Background:**

The spread of Covid-19 and resultant infection prevention strategies have disturbed the life routine of Chinese young adults, led to reduced physical activity (PA), prolonged screen time (ST) and inadequate sleep duration (SP), and made immense influence on their mental health (MH) and quality of life (QoL). E-Health literacy (EHL) can enable citizens to use available online information to respond to the highly complex information environment and make appropriate health decisions.

**Objective:**

This study aims to examine associations between adhering to 24-h movement (24HM) guidelines and MH and QoL among young adults, as well as to identify any mediating or moderating role of EHL in these associations.

**Methods:**

1742 young adults (20.03 ± 1.54 years old, 68.6% females) from north and south China completed self-report measures of 24HM (PA, ST and SP), health indicators (MH and QoL), EHL and demographic information through an online survey between 4 Apr and 16 Jun 2022. Generalized linear mixed models were applied for data analysis.

**Results:**

Results showed that adhering to PA, ST and SP guidelines were all positively connected with QoL while MH was associated with adhering ST or SP guidelines. Adhering to more of 24HM guidelines was linked to better MH and QoL. EHL significantly mediated the association of guideline adherence and QoL and moderate that of guideline adherence and MH.

**Conclusion:**

This is the first study to investigate the role of EHL on the associations between 24HM and MH as well as QoL during the Covid-19. The findings may contribute to further empirical research or intervention that aims to promote MH or QoL among young adults more effectively or provide valuable references for developing relevant strategies or policy of health promotion or public health events in China.

## Introduction

In order to control the coronavirus disease 2019 (Covid-19) swiftly, infection prevention strategies such as quarantine and travel limitation were performed across China (1). During the performance of these strategies, Chinese young adults had to go through the isolation in or outside of the campus while taking online courses (2). Most aspects of their lives were affected by quarantines, which subsequently lead to negative impacts on their mental health (MH) as well as quality of life (QoL) (3). For most Chinese young adults, entering college is the first chance to live independently (4), and they may not have been psychologically mature enough to face such a severe public health emergency (5). Additionally, young adults had especially high rates of internet usage (6) and were likely exposed to a multitude of information online that contributed to psychological distress because of the information overload (7). Moreover, the isolated environment limited the social activities (8), strengthened academic pressures (9), and increased the likelihood of psychological problems (10). Thus, the MH and QoL of Chinese young adults have deteriorated since the Covid-19 outbreak (11).

Prior studies have shown that Covid-19 has resulted in less physical activity (PA) (12, 13), more screen time (ST) (13, 14), and insufficient sleep (SP) (10, 14), and it is well-known that these could negatively influence people’s physical and mental health as well as QoL (10, 12–14). Importantly, individuals may carry out multiple health behaviors in a day (15), thus the health impact of these behaviors should be given integrated consideration. In line with this reasoning, the Canadian Society for Exercise Physiology (CSEP) created the premiere 24-h movement (24HM) recommendations for adults aged under 65, provided the “healthy threshold” for adult’s PA, ST and SP (16). While these guidelines were primarily based on the impact of 24HM on physical health outcomes (16), limited research examined the relationship between 24HM and MH as well as QoL (17).

As introduced before, associations may exist between 24HM and MH together with QoL. Since e-Health literacy (EHL) has effects on health behaviors, MH and QoL, it is reasonable to assume that EHL could play a role in the linkages of 24HM and MH together with QoL. Yet, it is uncertain whether EHL is a mediator or a moderator in these linkages. For the mediating effect, theoretically, EHL is an integrated set of knowledge-based context-specific capabilities which may show variation due to circumstances (18, 19). According to the daily experience, sleep quality may influence individuals’ capability of health-related communication, people who are more attached to screen behaviors may tend to avoid health advice with plenty of physical activities. Besides, more physical activities provide practitioners more chances to obtain health-related experience such as how to stretch and strengthen muscles. These cases imply that EHL does not remain constant and would be influenced by context. Thus, it is possible that 24HM may have impacts on EHL and indirectly influence MH and QoL. For the moderating effect, EHL may relieve the negative impacts of inadequate 24HM on MH or QoL. A recent study found that EHL may help safeguard MH and enhance QoL for those exhibiting Covid-19 symptoms (20).

EHL encompasses the skills to search for, comprehend, evaluate, and apply health information from digital sources in order to address a health issue or make health-related decisions (18). Due to social distancing protocols and increased demand for health resources during Covid-19, individuals are more prone to access health information electronically compared to before the pandemic (21, 22). EHL therefore becomes crucial. Previous research proved that adequate EHL is significantly linked healthy behavior engagement (19, 23, 24). Recently, scholars have also shown that EHL can have a positive effect on MH by protecting people from unreliable or poor-quality health information during the Covid-19 (21, 22, 25). Additionally, EHL represents individuals’ ability to cope with health problems and manage their health status via the electronic resources or tools, thus it is closely associated with QoL. While several studies have validated that EHL could significantly enhance MH (22, 25) and QoL (20, 26) during the pandemic, scarce research has analyzed the interrelationships between 24HM, EHL, MH, and QoL.

Given the above, the current study aims to: (1) examine adherence to 24HM guidelines in Chinese young adults; (2) investigate associations between meeting 24HM guidelines and MH as well as QoL; and (3) analyze the mediating and moderating roles of EHL in the connections between adherence to 24HM guidelines and MH/QoL, while controlling for key demographic factors. We hypothesized that (1) participants who met individual or the specific combination of two 24HM guidelines would potentially exhibit superior MH and QoL versus non-adherers; (2) meeting more 24HM guidelines would correlate with decreased MH symptoms and improved QoL; (3) EHL would be a salient mediator and moderator in the associations of the 24HM guidelines adherence and MH together with QoL.

## Methods

### Participants and design

A cross-sectional design as well as the convenience sampling were utilized (27). Participants were recruited from four colleges in Hebei and Fujian of China. The cities were selected based on contrasts in regional geography (northern vs. southern) and administrative level (provincial capital vs. prefecture). The issue of convenience and feasibility were also considered when selecting the target cities (28). The inclusion criteria required that young adults (≥18 years): (1) were fluent in Chinese, (2) provided informed consent and willingness to take part in the survey, (3) had no disabilities or disorders in their physical or mental function, and (4) had persisted in China after the onset of the Covid-19. This study received ethical approval from the Research Ethics Committee at Putian University, China.

### Procedures

The data collection was conducted with the help of lecturers from the target colleges from 4 Apr to 16 Jun 2022. The survey was administered after obtaining informed consent from participants. They were notified of their right to withdraw at any time. The questionnaire was delivered via a QR code linking to an online survey platform. Participants spent approximately 10 min completing the questionnaires.

### Measurements

Demographic information to obtain demographic information, the survey asked for gender, age, academic year, major, BMI, family economic status (monthly family income: RMB < 6,000; RMB 6000–10,000; RMB 10000–14,000; RMB 14000–18,000; RMB > 18,000), parents’ highest education level, and geographical region.

Physical activity (PA) was assessed by the abridged Chinese edition of the International Physical Activity Questionnaire (IPAQ-C) [International Physical Activity Questionnaire, (29); IPAQ-C, (30)], and a self-designed question, asking participants how many times of muscle strengthen activities they have performed per week. The IPAQ-C comprises 6 items assessing self-reported PA across three intensities (vigorous, moderate, mild) over the past week. For each intensity, participants indicated how many days per week and how many minutes per day they took part in the activities. Total weekly PA time and time in each intensity category (minutes/week) were calculated based on responses (31). The 24HM Guidelines (16) define meeting PA recommendations as ≥150 min weekly of moderate-to-vigorous PA (MVPA) plus twice weekly muscle strengthening (16).

Sleep (SP) was measured by three items extracted from the Pittsburgh Sleep Quality Index (PSQI) (32). Questions include: “Thinking about the previous week, what were your average wake-up and bedtimes each day? How many hours did you sleep nightly on average?” Meeting SP recommendations per the 24HM guidelines (16) requires 7–9 h daily.

Screen time (ST) was measured using the Chinese translated Sedentary Behavioral Questionnaire (33). Six questions were asked: “Considering the last 7 days, what was your mean time allotted on weekdays/weekends for activities such as videos, gaming, chatting, web searches, email, and studying?” The 24HM Guidelines (16) recommend limiting ST time to ≤8 h daily.

Mental health (MH) was quantified via the 21-question Chinese ‘Depression Anxiety Stress Scale’ (DASS-21) (34, 35), which has demonstrated validity among the adult mainland Chinese population. The 21-item DASS comprises three 7-item subscales assessing participants’ feeling of depression, anxiety and stress regarding the previous week (Cronbach’s *α* = 0.79–0.89). Each item uses a 4-point response format from 0 (“irrelevant to me”) to 3 (“extremely relevant to me”). Subscale values are obtained by totaling the item responses and doubling the sum. Cutoffs classify depression as normal (0–9) or symptomatic (>9), anxiety as normal (0–7) or symptomatic (>8), and stress as normal (0–14) or symptomatic (>15). Symptomatic levels include mild, moderate, severe and extremely severe (36).

Quality of life (QoL) was measured utilizing the brief Chinese edition of the World Health Organization Quality of Life scale (37) (Cronbach’s *α* = 0.82). Participants rated their perceptions over the previous 4 weeks. They firstly measured their overall QoL with a 5-point scale from 1 (“very poor”) to 5 (“very good”), then completed 7 items evaluating satisfaction with physical health subdomains on a 5-point scale from 1 (“very dissatisfied”) to 5 (“very satisfied”).

e-Health literacy (EHL) was assessed using the e-Health literacy scale in Web 3.0 context (eHLS-Web3.0) developed by Liu et al. (38), consisting of 24 items assessing EHL across three dimensions: acquisition, verification and application utilizing a 5-point scale from 1 (strongly disagree) to 5 (strongly agree). The tool demonstrates validity via exploratory and confirmatory factor analyses. Its reliability, validity, and measurement invariance have been established among young adult populations. Compared to previous EHL measures, the eHLS-Web3.0 captures modern technological engagement including social media and mobile internet. Recently developed, it shows high internal consistency for the overall scale (*α* = 0.971) and subscales (*α* = 0.913–0.962) in Chinese young adults.

### Data analysis

IBM SPSS 27.0 (Armonk, NY) facilitated data analyses. Initial diagnostics were conducted including outlier and distribution analyses. Skewed variables were log-transformed or replaced with median (interquartile range). Participant characteristics were summarized using descriptive statistics: mean, standard deviation (SD), and frequencies (%). Generalized linear mixed models were used to examine the relationships of meeting the 24HM guidelines with MH and QoL, controlling for all demographic covariates (i.e., gender, age, year of study, major, BMI, family economic status, parents’ education levels and region).^1^ The sample size was sufficient to detect an effect size of (Cohen *f*^2^) 0.01, with an alpha of 0.05 and 1-β of 0.8 in the regression model (42). A two-tailed *p*-value under 0.05 denoted statistical significance. Model 1 and Model 4 of PROCESS was utilized in SPSS to test the mediating and moderating effect of e-Health literacy. To confirm the significance of the indirect effect, bias-corrected bootstrapped of 95% confidence intervals with 5,000 resamples were also applied. We established statistical significance as *p* < 0.05 (two-tailed). Effect sizes for model predictors were calculated using Cohen’s *f*^2^, computed by the formula “*f*^2^ = *R^2^*/(1-*R*^2^).” Values of 0.02, 0.15, and 0.35 indicate small, medium and large effects, respectively (43).

## Results

### Demographic characteristics and guideline adherence

A summary of participants’ demographic information and guideline adherence are described in Table 1. Out of 1,862 young adults approached, 1,850 gave written informed consent to participate. After eligibility check and data screening, the final analytical sample comprised 1,742 participants. The sample comprised 547 males and 1,195 females, aged 18 to 25 years (*M* = 20.03, SD = 1.54). A considerable proportion of the sample reported a health-related major (43.3%). In terms of body mass index (BMI, kg/m^2^), most of the participants were in a healthy level. 44.4% of participants reported meeting the PA guideline, 80.3% meeting ST, and 78.9% meeting SP. Percentages of participants adhering to a combination of two 24HM guidelines ranged from 35 to 63.6%. 28.5% of participants meet all the 24HM recommendations. An adequate level of EHL (*M* = 3.73, SD = 0.68) was demonstrated among the participants.

**Table 1:** Participants’ characteristics and 24-h guideline adherence.

Demographic information	Value (*n* = 1742)
Age (Years), mean (SD)	20.03 (1.54)
Gender (female, %)	1,195, 68.6%
Region (*n*, %)	
North	627, 36%
South	1,115, 64%
Monthly family income (*n*, %)	
RMB < 6,000	554, 31.8%
RMB 6000–10,000	494, 28.4%
RMB 10000–14,000	148, 8.5%
RMB 14000–18,000	36, 2.1%
RMB > 18,000	48, 2.8%
Not sure	462, 26.5%
Parents education (*n*, %)	
Below primary school	464, 26.6%
High school	1,028, 59%
Diploma	133, 7.6%
College or above	117, 6.7%
Major (*n*, %)	
Sports-related	165, 9.5%
Medical-related	590, 33.9%
Non-health related	987, 56.7%
BMI (*n*, %)	
Underweight	378, 21.7%
Health	1,146, 65.8%
Overweight	218, 12.5%
E-health literacy, mean (SD)	3.73 (0.68)
Guideline adherence (*n*, %)	
Meeting PA	774, 44.4%
Meeting ST	1,398, 80.3%
Meeting SP	1,374, 78.9%
Meeting PA + ST	610, 35%
Meeting PA + SP	629, 36.1%
Meeting ST + SP	1,108, 63.6%
Meeting PA + ST + SP	497, 28.5%

BMI, body mass index (kg/m2); SD, standard deviation; PA, physical activity; ST, screen time; SP, sleep.

### 24HM guideline adherence and health outcomes (MH and QoL)

Table 2 displays the links between compliance with guidelines and health indicators (individual and in combination). After adjusting the covariates (i.e., gender, age, year of study, major, BMI, family economic status, parents’ education levels and region), meeting the guideline of PA was associated with QoL (*β* = 0.176, 95%CI = 0.114, 0.239); meeting the guideline of ST was associated with QoL (*β* = 0.092, 95%CI = 0.017, 0.166) and lower depression symptoms (*β* = −1.872, 95%CI = -2.441, −1.304), anxiety (*β* = −1.582, 95%CI = -2.138, −1.027) and stress (*β* = −1.576, 95%CI = -2.154, −0.99); meeting the guideline of SP was associated with QoL (*β* = 0.280, 95%CI = 0.208, 0.353), as well as lower stress (*β* = −1.102, 95%CI = -1.666, −0.538), depression symptoms (*β* = −0.595, 95%CI = -1.149, −0.041) and anxiety (*β* = −0.842, 95%CI = -1.384, −0.300) (all *p* < 0.05).

**Table 2:** Associations between guideline adherence and stress, depression, anxiety and quality of life.

Meet guidelines	Depression		Anxiety		Stress		Quality of Life	
	Multivariate model	Adjusted model	Multivariate model	Adjusted model	Multivariate model	Adjusted model	Multivariate model	Adjusted model
	β(95% CI)	β(95% CI)	β(95% CI)	β(95% CI)	β(95% CI)	β(95% CI)	β(95% CI)	β(95% CI)
Meeting (*vs* not meeting) individual guideline								
At least PA	0.102 (−0.381 ~ 0.585)	0.114 (−0.363 ~ 0.592)	0.150 (−0.322 ~ 0.622)	0.173 (−0.294 ~ 0.640)	0.070 (−0.422 ~ 0.562)	0.103 (−0.383 ~ 0.589)	0.187 (0.123 ~ 0.250)	0.176 (0.114 ~ 0.239)
*P*	0.679	0.639	0.534	0.467	0.782	0.678	<0.001	<0.001
At least ST	−1.883 (−2.451 ~ −1.314)	−1.872 (−2.441 ~ −1.304)	−1.597 (−2.154 ~ −1.040)	−1.582 (−2.138 ~ −1.027)	−1.594 (−2.175 ~ −1.012)	−1.576 (−2.154 ~ −0.99)	0.095 (0.018 ~ 0.171)	0.092 (0.017 ~ 0.166)
*P*	<0.001	<0.001	<0.001	<0.001	<0.001	<0.001	0.015	0.016
At least SP	−0.616 (−1.176 ~ −0.057)	−0.595 (−1.149 ~ −0.041)	−0.857 (−1.403 ~ −0.310)	−0.842 (−1.384 ~ −0.300)	−1.120 (−1.688 ~ −0.552)	−1.102 (−1.666 ~ −0.538)	0.291 (0.218 ~ 0.364)	0.280 (0.208 ~ 0.353)
*P*	0.031	0.035	0.002	0.002	<0.001	<0.001	<0.001	<0.001
Meeting (*vs* not meeting) specific combination								
At least PA + ST	−0.456 (−0.945 ~ 0.033)	−0.521 (−1.185 ~ 0.144)	−0.335 (−0.813 ~ 0.143)	−0.216 (−0.865 ~ 0.433)	−0.381 (−0.879 ~ 0.118)	−0.078 (−0.753 ~ 0.597)	0.179 (0.115 ~ 0.244)	0.012 (−0.075 ~ 0.100)
*P*	0.068	0.125	0.17	0.513	0.134	0.821	<0.001	0.78
At least PA + SP	−0.015 (−0.511 ~ 0.481)	0.712 (0.045 ~ 1.380)	−0.074 (−0.558 ~ 0.411)	0.459 (−0.193 ~ 1.111)	−0.229 (−0.733 ~ 0.276)	0.251 (−0.427 ~ 0.929)	0.233 (0.168 ~ 0.298)	0.183 (0.095 ~ 0.271)
*P*	0.953	0.036	0.765	0.167	0.375	0.468	<0.001	<0.001
At least ST + SP	−1.362 (−1.834 ~ −0.890)	−1.392 (−1.886 ~ −0.898)	−1.389 (−1.849 ~ −0.928)	−1.442 (−1.924 ~ −0.959)	−1.556 (−2.035 ~ −1.076)	−1.596 (−2.098 ~ −1.094)	0.202 (0.139 ~ 0.264)	0.153 (0.088 ~ 0.218)
*P*	<0.001	<0.001	<0.001	<0.001	<0.001	<0.001	<0.001	<0.001
Number of guidelines met								
Meet none	Reference	–	Reference	–	Reference	–	Reference	–
Meet one	−2.032 (−3.514 ~ −0.550)	–	−1.542 (−2.991 ~ −0.093)	–	−1.518 (−3.089 ~ −0.074)	–	0.388 (0.195 ~ 0.582)	–
*P*	0.007		0.037		0.04		<0.001	
Meet two	−2.835 (−4.262 ~ −1.408)	–	−2.406 (−3.801 ~ −1.011)	–	−2.598 (−4.050 ~ −1.146)	–	0.553 (0.366 ~ 0.739)	–
*P*	<0.001		0.001		<0.001		<0.001	
Meet three	−3.085 (−4.542 ~ −1.627)	–	−2.638 (−4.062 ~ −1.213)	–	−2.909 (−4.392 ~ −1.426)	–	0.716 (0.526 ~ 0.907)	–
*P*	<0.001		<0.001		<0.001		<0.001	

95%CI, 95% confidence interval; PA, physical activity; ST, screen time; SP, sleep. Multivariate model: Bi-variable association between guideline adherence and health outcomes adjusting for covariates. Adjusted model: Adjusted for covariates and adherence of other guidelines.

Meeting PA + ST has no significant association with mental health or quality of life. Meeting PA + SP guideline was associated with depression symptoms (*β* = 0.712, 95%CI = 0.045, 1.380) and QoL (*β* = 0.183, 95%CI = 0.095, 0.271); meeting ST + SP guidelines was connected to relieved depression (*β* = −1.392, 95%CI = -1.886, −0.898), anxiety (*β* = −1.442, 95%CI = -1.924, −0.959) and stress (*β* = −1.596, 95%CI = -2.098, −1.094) symptoms, and higher QoL (*β* = 0.153, 95%CI = 0.088, 0.218). Meeting all guidelines was related to higher level of QoL (*β* = 0.716, 95%CI = 0.526, 0.907) and lower symptoms of depression (*β* = −3.085, 95%CI = -4.542, −1.627), anxiety (*β* = −2.638, 95%CI = -4.062, −1.213) and stress (*β* = −2.909, 95%CI = -4.392, −1.426).

### The mediating and moderation effects of EHL

A significant moderating effect of EHL emerged in the analysis between adhering to guidelines and stress, depression, and anxiety symptoms (see Table 3). The moderation effect of EHL on the association of guideline adherence and QoL was not significant. Regarding the association between guideline adherence and stress, significant interactions emerged between EHL and meeting one guideline (*β* = 0.374, *p* = 0.015) as well as EHL and meeting two guidelines (*β* = 0.334, *p* = 0.024). The interactions contributed a significant change in the explained variance (△*R*^2^ = 0.007, *p* < 0.01). Overall, the moderation model explained 8% of variance in stress scores (*R*^2^ = 0.08, *p* < 0.001). On the association between guideline adherence and depression, results indicated a significant interaction between EHL and adhering to one guideline (*β* = 0.344, *p* = 0.025). The interaction contributed to a significant change in the explained variance (△*R*^2^ = 0.006, *p* = 0.010). Overall, the moderation model explained 8% of variance in depression scores (*R*^2^ = 0.08, *p* < 0.001). On the association between guideline adherence and anxiety, the interaction between EHL and compliance with one guideline (β = 0.322, *p* = 0.037) contributed to a significant change in the explained variance (△*R*^2^ = 0.007, *p* = 0.007). Total variance explained in anxiety was 7% (*R*^2^ = 0.07, *p* < 0.001). For students adhering to more behavioral guidelines, the moderating effect of EHL was mild, whereas for those adhering fewer behavioral guideline, a higher level of EHL was associated with the negative impact of health-compromising behavior and mental health.

**Table 3:** Moderating effect of e-Health literacy on the association between guideline adherence and stress, depression, anxiety and quality of life (*N* = 1,742).

Model	Stress		Depression		Anxiety		Quality of Life	
	Coefficient value	*p*	Coefficient value	*p*	Coefficient value	*p*	Coefficient value	*p*
X1	−0.159	0.339	−0.272	0.101	−0.203	0.226	0.404	0.007
X2	−0.369	0.022	−0.443	0.006	−0.392	0.016	0.594	0
X3	−0.412	0.012	−0.48	0.003	−0.421	0.011	0.796	0
EHL	−0.322	0.026	−0.289	0.045	−0.248	0.088	0.523	0
X1*EHL	0.374	0.015	0.344	0.025	0.322	0.037	−0.108	0.437
X2*EHL	0.334	0.024	0.282	0.057	0.27	0.07	−0.12	0.373
X3*EHL	0.196	0.194	0.16	0.286	0.131	0.388	−0.062	0.652
△R^2^	0.007	0.005	0.006	0.01	0.007	0.007	0.001	0.574

All models were adjusted for gender, age, year of study, major, BMI, family economic status, parents’ education levels and region; X1, meeting one behavioral guideline; X2, meeting two behavioral guidelines; X3, meeting three behavioral guidelines. EHL, e-Health literacy. △R2, change in the variance explanation. X1/2/3*EHL, interaction between EHL and adhering 1/2/3 guideline(s).

Table 4 shows the results of the mediation analyses conducted to determine whether guideline adherence predicted stress, depression, anxiety and QoL through EHL. The relationships between EHL and anxiety/depression/stress were not significant (β_anxiety_ = −0.016, *p* = 0.503; β_depression_ = −0.038, *p* = 0.102; β_stress_ = −0.029, *p* = 0.215), indicating that EHL did not mediate the relationship between guideline adherence and mental health. The mediating effect of EHL was found for the association between guideline adherence and QoL, no matter how many guidelines were complied. Further, the direct effect of guideline adherence on QoL (c’) remained significant after EHL and other covariates were controlled for, indicating a partial mediation of EHL. The total mediation model accounted for 25% of the variance in QoL (R^2^ = 0.25, *p* < 0.001).

**Table 4:** Mediating effect of e-Health literacy on the association between guideline adherence and stress, depression, anxiety and quality of life (*N* = 1,742).

Model		X1		X2		X3	
Outcome variable	Interaction	Coefficient value	*p*	Coefficient value	*p*	Coefficient value	*p*
Anxiety	a	0.34	0.029	0.49	0.001	0.613	0
	b	−0.016	0.503	−0.016	0.503	−0.016	0.503
	c’	−0.323	0.035	−0.5	0.001	−0.541	0
	c	−0.328	0.032	−0.508	0.001	−0.551	0
Depression	a	0.34	0.029	0.49	0.001	0.613	0
	b	−0.038	0.102	−0.038	0.102	−0.038	0.102
	c’	−0.402	0.008	−0.559	0	−0.607	0
	c	−0.415	0.006	−0.578	0	−0.631	0
Stress	a	0.34	0.029	0.49	0.001	0.613	0
	b	−0.029	0.215	−0.029	0.215	−0.029	0.215
	c’	−0.306	0.045	−0.505	0.001	−0.559	0
	c	−0.316	0.038	−0.519	0	−0.577	0
Quality of life	a	0.34	0.029	0.49	0.001	0.613	0
	b	0.425	0	0.425	0	0.425	0
	c’	0.45	0.001	0.639	0	0.845	0
	c	0.595	0	0.848	0	1.106	0

All models were adjusted for gender, age, year of study, major, BMI, family economic status, parents’ education levels and region; X1, meeting one behavioral guideline; X2, meeting two behavioral guidelines; X3, meeting three behavioral guidelines. a, interaction between e-Health literacy and guideline adherence; b, interaction between e-Health literacy and outcome variables; c’, indirect interaction between guideline adherence and outcome variables; c, direct interaction between guideline adherence and outcome variables.

## Discussion

This research analyzed adherence to recommendations for 24HM among Chinese university students, examined the relationship between guideline adherence and MH and QoL, and identified the mediating or moderating role of EHL. Findings showed that meeting PA, ST and SP guidelines were all positively connected with QoL while MH was associated with meeting ST or SP guidelines. Furthermore, adhering to more 24HM guidelines was associated with improved MH and QoL, but meeting different combinations of guidelines also led to different relationships with MH and QoL. EHL is also found to be a mediator of the relationship between guideline adherence and QoL and a moderator of the relationship between guideline adherence and MH.

Among the three movement guidelines, the PA guideline had the lowest compliance rate. This might be because the study was conducted in April 2022, during the outbreak of Omicron in China. Omicron is one of the most easily spread variants of Covid-19 (44) and some infection prevention strategies were taken by universities during this time, including entry-school restrictions, social distancing and lockdown inside school (2). The adherence to the PA guidelines might have been influenced by pandemic restrictions. Still, compared to previous research (17, 45, 46), the compliance in the current study was higher. The possible reason is that the participants are all college students living on campus, nearly half of whom (43.3%) where from health-related major. Life on campus is usually comprised of a regular routine, and students’ health behaviors may be positively influenced by their interests in their health major and their peers. Environmental factors such as peer support or perceived social norms should be considered in further relevant studies.

Meeting ST and SP guidelines were associated with better MH and QoL, which partially supported our hypothesis. However, a linkage was not significant between PA guideline adherence and mental health. This unexpected result is inconsistent with previous research that has established PA as an effective therapy for mental illness symptoms (47, 48). However, several articles reported a similar finding with the current study (49–52). A review on PA intervention among young people demonstrated that there was limited evidence for PA intervention to benefit mental illness (53). Consistent with the current study, research by Walsh et al. (49) and Guerrero et al. (50) showed no link between adherence to PA recommendations and better MH among American youth. Rebar et al. (54) suggested that the effect of PA on MH was smaller among non-clinical groups than the clinical ones, which might explain this finding. The sample characters in this study may have contribute to the lack significant association with PA. Additionally, most college students were required to take PE courses, and on-campus compulsory quarantine in April 2022 (2) also required students to take extra walk to arrive the quarantine station at least three times per week. These ‘compulsory tasks’ may also limit the positive effect of PA on MH observed in a more autonomous situation.

Consistent with hypothesis 2, increased compliance with guidelines corresponded to better MH and QoL outcomes. Meanwhile, meeting different combinations of guidelines had different effects on students’ MH and QoL. Among the three combinations (PA + ST, PA + SP, ST + SP), meeting ST + SP guidelines showed the most significant positive effects on both MH and QoL, which is consistent with previous studies (45, 46). Surprisingly, meeting PA + SP guideline was found to increase the symptoms of depression with a moderate effect size. This result might again be another demonstration of the negative impact of unhealthy ST on depression during Covid-19. There were infection peaks in April 2022 because of the spread of Omicron and subsequently an overwhelming influx of information about emerging infectious disease was booming online. For students who were having a lockdown inside the school, the internet was the main channel for them to learn about the situation outside (55). The enormous amount of information and messages about the infection may have created a huge challenge to their mental health (21). Additionally, meeting PA + ST had no significant impact on either MH or QoL. Several previous studies have highlighted the relative importance of meeting SP guideline (45, 46, 56), indicated that the SP guideline had a stronger connection to MH compared to the other guidelines. It is possible that the negative impact of not meeting SP guideline inhibits the positive effect of meeting PA + ST guideline. Furthermore, the mandatory PA may also be limiting the findings of PA in this population. Overall, the findings of this study reveal that PA, ST and SP are three codependent behaviors which may impact each other and occupy each other’s time within 24 h. Future research should continue to consider the differences in these combinations when implementing behavioral interventions, and priority should be given to which behavioral guideline should also be further studied so as to provide greater impact on various health benefits.

As far as we know, this is the first cross-sectional study examining the role of EHL in the relationship between 24HM and MH as well as QoL among Chinese young adults during the Covid-19. Prior studies have discussed various possibilities for how health literacy links to health outcomes (57, 58), although actual research on these mechanisms is scant. EHL is a concept developed on the basis of health literacy (18) and the mechanism of it influencing health might be similar. Health literacy is frequently found as a mediator or a moderator of the relationship between other determinants and health (57). The results of this study support EHL as a mediator between guideline adherence and QoL, and a moderator between guideline adherence and MH. Overall these findings thus partially support hypotheses 3. Health literacy has long been seen as a mediating factor between behavior and health because it has a potentially adaptable impact on social factors determining health outcomes (58). As a key element of public health surveillance, QoL had long be seen as an integrated predictor for individual’s perceived physical, mental and social health (59). This result supports the intervening effect of EHL on social-life-related QoL, proposing that improve EHL can potentially strengthen the positive health impacts of adhering behavioral guidelines. It is worthwhile to explore the relationship between 24HM and QoL among young adults during the Covid-19, which could help obtain a whole picture for the health impact of 24HM guideline.

In terms of the moderating effect, EHL was found to be a stronger moderator among students who were adhering to less guidelines. This finding might have been because that students adhering to more behavioral guidelines had better competency in health management and perceive better controllability when facing health threats (i.e., Covid-19) (60). However, for students adhering less to guidelines, they may have had limited health-managing personal experiences to draw upon, and had to rely on messages mainly from the internet. Among this group, EHL may substantially influence their understanding of such messages, thus creating stronger moderating effect between behavioral guidelines and MH. A Romanian study also found EHL acted as a protective factor against emotional distress related to searching for health information online (61). This finding indicates the necessity of providing EHL workshop or intervention for young adults, especially for those from non-health-related majors.

### Limitations

Despite this study’s innovative and interesting findings, some limitations exist. First, the cross-sectional design of this study precludes the causality assumptions, and a reverse causation might be equally plausible. Further longitudinal research is needed to confirm these results. Second, the use of self-report measures may have contributed to recall bias, as well as the over/under-reporting and social desirability biases common in surveys. Further, the ST measured in this study was not limited to ‘recreational’ or ‘non-recreational’ ST; therefore, potential inaccuracies in the adherence of ST guideline might occurred. Third, the data collection was conducted during an infection peak of Omicron, so the timing may have influenced participants’ movement behaviors compared to other situations. Fourth, this study applied an online convenience sampling method, which may lead to some selection bias, despite the large sample. Finally, although the focus of this research was on the role of EHL and the relationship between movement guideline adherence and QoL/MH, there may still be other potential mediators that have not been taken into consideration. Continued exploration of key mechanisms linking movement behaviors to QOL and MH are warranted.

## Conclusion

The current study examined the relationship between adhering to the 24HM guidelines and MH/QoL during Covid-19 and identified the effect of EHL in mediating the 24HM-QoL association and moderating the 24HM-MH association. It was found that meeting more of the guidelines was related to better MH and QoL among Chinese young adults, while meeting different combinations of the guidelines led to different health outcomes. The findings of this study highlighted the importance of EHL, offered new insights for implementing more effective MH and QoL interventions in the future, and supported guidance of 24HM guidelines when understanding health behaviors.

## Data availability statement

The raw data supporting the conclusions of this article will be made available by the authors, without undue reservation.

## Ethics statement

The studies involving humans were approved by The Research Ethics Committee at Putian University, China. The studies were conducted in accordance with the local legislation and institutional requirements. The participants provided their written informed consent to participate in this study. Written informed consent was obtained from the individual(s) for the publication of any potentially identifiable images or data included in this article.

## Author contributions

LL: Writing – review & editing, Supervision, Resources, Project administration. WL: Writing – review & editing, Writing – original draft, Validation, Resources, Project administration, Methodology, Investigation, Data curation. RW: Writing – review & editing, Supervision, Project administration. RR: Writing – review & editing, Validation, Supervision. HL: Writing – review & editing, Writing – original draft, Validation, Project administration, Investigation, Data curation.

## Footnotes

^1^Previous research demonstrated that these variables were associate with people’s 24-h movement, e-Health literacy and diverse health outcomes (39–41).
